# Rbp4-Gal4, a germline driver that activates in meiosis, reveals functions for VCP in spermatid development

**DOI:** 10.1080/19336934.2023.2234795

**Published:** 2023-07-12

**Authors:** Tyler J. Butsch, Alyssa E. Johnson, K. Adam Bohnert

**Affiliations:** Department of Biological Sciences, Louisiana State University 202 Life Sciences Building, Baton Rouge, LA, USA

**Keywords:** germline, male fertility, spermatogenesis, spermatocyte, spermatid, VCP, individualization, Gal4/UAS

## Abstract

Valosin-containing protein (VCP) is a versatile and ubiquitously expressed AAA+ ATPase that regulates multiple stages of *Drosophila* spermatogenesis. While VCP has documented roles in mitotic spermatogonia and meiotic spermatocytes, it is also highly expressed in post-meiotic spermatids, suggesting potential late-stage developmental functions as well. However, tools to assess late-stage activities of pleiotropic spermatogenesis genes such as *VCP* are lacking. Available germline-specific Gal4 drivers activate in stem cells or spermatogonia; consequently, knocking down *VCP* using one of these drivers disrupts or blocks early germ-cell development, precluding analysis of VCP in later stages. A Gal4 driver that activates later in development, such as at the meiotic spermatocyte stage, may permit functional analyses of VCP and other factors in post-meiotic stages. Here, we describe a germline-specific Gal4 driver, Rbp4-Gal4, which drives transgene expression beginning in the early spermatocyte stage. We find that Rbp4-Gal4-driven knockdown of *VCP* causes defects in spermatid chromatin condensation and individualization without affecting earlier developmental stages. Interestingly, the defect in chromatin condensation appears linked to errors in the histone-to-protamine transition, a key event in spermatid development. Overall, our study reveals roles for VCP in spermatid development and establishes a powerful tool to dissect the functions of pleiotropic spermatogenesis genes.

## Introduction

**Figure 1.**
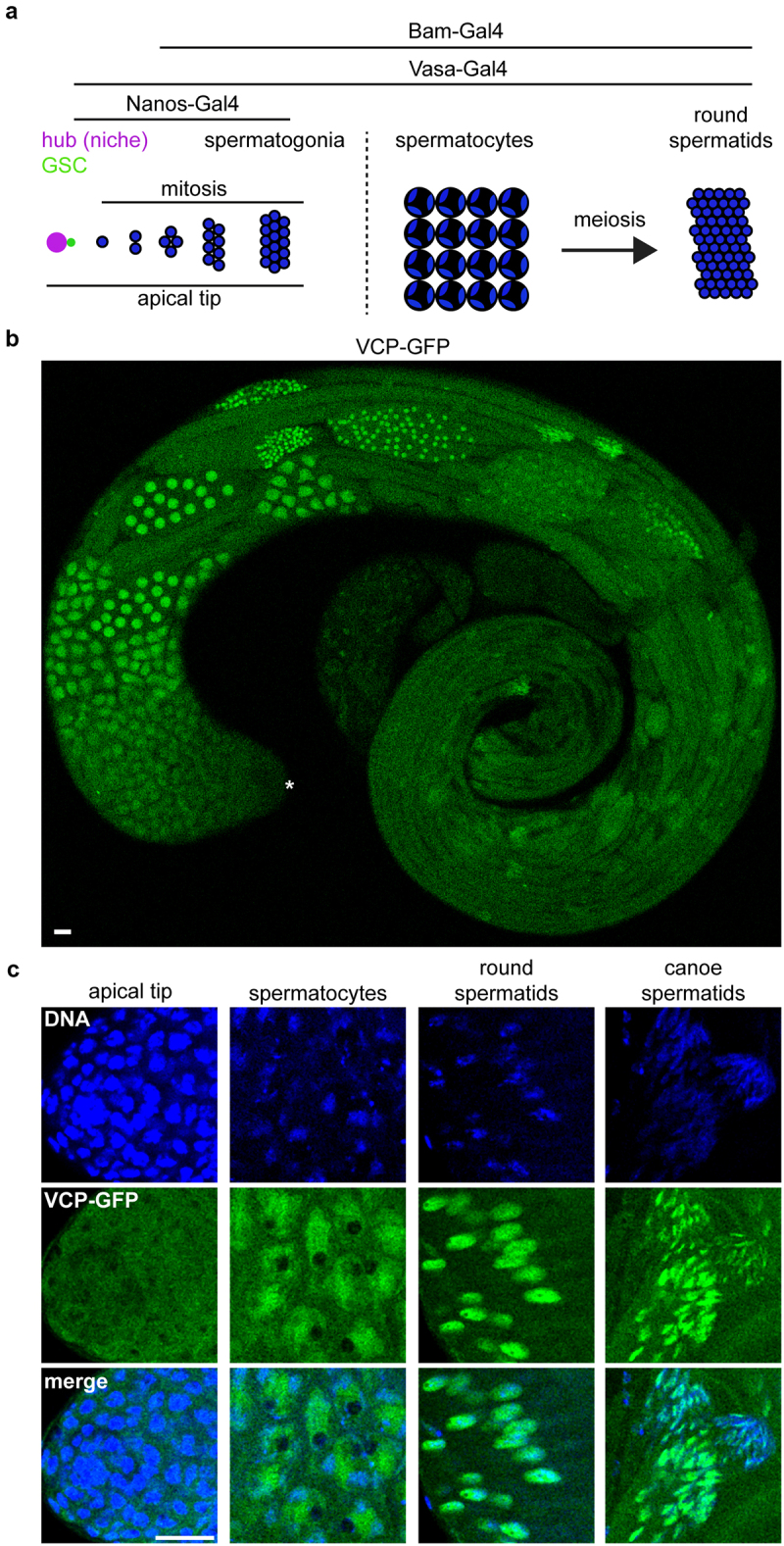
VCP is expressed throughout the male *Drosophila* germline. (a) Schematic of *Drosophila* spermatogenesis. Stages where Nanos-Gal4, Vasa-Gal4 and Bam-Gal4 are active are indicated. (b) Low magnification images of a whole VCP-GFP testis. The asterisk indicates the apical tip of the testis, where spermatogenesis initiates. (c) Images of Hoechst (DNA) and VCP-GFP in spermatogonia, spermatocytes, round spermatids and canoe-stage spermatids. Bars, 20 µm.

Pleiotropy is the ability of a gene to serve multiple functions, sometimes even in the same biological process. One such example is Valosin-containing protein (VCP, also known as TER94), a AAA+ ATPase that regulates diverse biological processes [1], including multiple stages of *Drosophila* spermatogenesis. During the initial stages of *Drosophila* spermatogenesis, germline stem cells (GSCs) give rise to mitotic spermatogonia, which then differentiate into meiotic spermatocytes (Figure 1a). When *VCP* is genetically knocked down specifically at the start of spermatogenesis, mitotic spermatogonia hyper-proliferate [2], suggesting that VCP regulates developmental timing early in spermatogenesis. Recently, we discovered that VCP performs an additional, essential function during meiosis; VCP downregulates a repressive histone modification, mono-ubiquitinated H2A (H2Aub), to promote spermatocyte differentiation, and experimentally blocking this activity causes an early meiotic arrest [3]. Thus, *VCP* is a pleiotropic spermatogenesis gene that executes separable functions at distinct developmental timepoints and inhibition of VCP function in the testis can yield different phenotypes depending on the precise stage of inhibition.

While previous findings indicate at least two separable functions of VCP during spermatogenesis, it remains possible that VCP may exert even more activities during this process, perhaps even at later stages. Following meiotic divisions, spermatocytes differentiate into spermatids (Figure 1a). During the spermatid stage, germ cells elongate and histones are replaced by arginine-rich proteins known as protamines, driving hyper-condensation of chromatin to ultimately facilitate the formation of mature sperm [4–6]. Interestingly, a large-scale mutagenesis analysis [7] identified a male-sterile allele of *VCP* (FBal0325644) that is reported to cause defects at the spermatid stage (personal communication to Flybase; FBrf0235035). Yet, this mutation is present in all cell types, and it is unclear whether the associated defects are due to *VCP* dysfunction in the soma or germline. Potentially, tools to control VCP activity in a germline- and stage-specific manner could help to clarify the extent to which VCP regulates previously unlinked events in spermatogenesis, including at the spermatid stage.

Gene function in *Drosophila* spermatogenesis is commonly manipulated using the Gal4/UAS system [8,9], a powerful genetic toolkit that permits spatiotemporal control of transgene expression [10,11]. Currently, there are three main Gal4 drivers used to study gene function in the male germline: Vasa-Gal4, Nanos-Gal4 and Bam-Gal4 [8]. Vasa-Gal4 becomes active in GSCs and maintains activity throughout the male germline (Figure 1a). Nanos-Gal4 is also active in GSCs but exhibits minimal activity outside of GSCs (Figure 1a). Bam-Gal4 becomes active in spermatogonia but also supports transgene expression later in development, such as in spermatocytes and post-meiotic round spermatids (Figure 1a). A germline-specific Gal4 driver that consistently and robustly activates in meiotic-stage germ cells (i.e. following the completion of mitotic stages) has, to our knowledge, not been reported. Researchers have previously attempted to control transgene expression in meiotic stages of spermatogenesis using the *β2-tubulin* promoter, which activates late in the spermatocyte stage [9]. However, the generation of functional Gal4 lines under control of the *β2-tubulin* promoter has been unsuccessful, possibly because the promoter activates too far along in development to drive sufficient Gal4 expression [9]. The lack of a driver specific to post-spermatogonial stages of *Drosophila* spermatogenesis has hindered the study of pleiotropic spermatogenesis genes at later timepoints in germ-cell development. In the case of *VCP*, RNAi-mediated knockdown using one of the three aforementioned Gal4 drivers causes a developmental abnormality or arrest early in germ-cell development [2,3], abrogating functional analysis later in spermatogenesis. Accordingly, a germline-specific Gal4 driver that activates developmentally later than Bam-Gal4 could be of use in deciphering later-stage functions of *VCP* and possibly other pleiotropic spermatogenesis genes.

In this study, we designed a Gal4 driver under the control of the promoter of *Rbp4*, a germline-specific gene that is strongly expressed following the completion of mitosis (Fig. S1) [12–14]. We find that Rbp4-Gal4 is inactive until germ cells enter the spermatocyte stage; thus, it can be used to effectively drive transgene expression in germ cells that have progressed past the spermatogonial stage of spermatogenesis. When used to drive *VCP-*RNAi, Rbp4-Gal4 reliably knocks down *VCP* expression in both spermatocytes and spermatids. Interestingly, *VCP* knockdown driven by Rbp4-Gal4 reveals a previously unknown, germline-specific requirement for VCP in spermatid chromatin condensation and individualization. Intriguingly, our data also suggest that VCP promotes the histone-to-protamine transition in developing spermatids. Overall, our study establishes a tool that enables the analysis of later-stage functions of pleiotropic spermatogenesis genes, using *VCP* as a proof-of-principle.

## Materials and methods

### Fly husbandry and strains

Flies were maintained on standard cornmeal/agar food at 25°C, unless otherwise noted. For RNAi experiments, flies were incubated on standard cornmeal/agar food supplemented with yeast paste at 29°C for 7 d prior to dissection and imaging to boost Gal4 activity and gene knockdown.

The following fly strains were used in this study: w^1118^ (lab stock), Bam-Gal4 (Doug Harrison, Univ. Kentucky), VCP-sfGFP [15], UAS-bam-RNAi (BDSC #33631), protB-GFP (outcrossed from BDSC #58406), UAS-VCP-RNAi (VDRC #24354) and UAS-GFP^nls^ (lab stock).

### Identification of candidate promoters

To identify an appropriate spermatocyte-specific promoter, we mined previously published gene expression datasets to identify candidate genes with spermatocyte-specific gene expression profiles. First, we used the dataset from [14] to identify candidate genes that were lowly expressed in spermatogonia but highly expressed throughout the spermatocyte stage. This was done by dividing RPKM expression values at the early spermatocyte stage by expression values at the 16-cell spermatogonia stage (EC/S16). We filtered out genes with an EC/S16 less than 10, RPKM above 50 at any spermatogonial stage, and any gene symbol beginning with ‘CG’ or ‘CR’ (unannotated genes), which resulted in a list of approximately 5000 genes. We then cross-referenced the selected genes with genes that were specifically mentioned to be expressed in spermatocytes in an independent RNAseq study [13] and other genes we were familiar with from previous studies, such as *hsc70–2* [16], *mst35Bb* [5] and *htrA2* [17]. To this point, we had identified 22 candidate genes that are highly expressed in spermatocytes and lowly expressed in spermatogonia. Finally, we cross-referenced this list to FlyAtlas [18,19] to select against genes that were expressed in somatic tissues, which resulted in a final list of 15 genes. Following this analysis, we sorted genes from highest to lowest EC value. The top gene was *Rbp4* (Fig. S1).

### Construction of Rbp4-Gal4

We used a Gateway cloning toolkit [20] and associated enzymes for all cloning steps. We first amplified the putative *Rbp4* promoter by amplifying a 2.3kb region immediately upstream of the *Rbp4* coding region (*pRbp4*) from genomic DNA using the following primers:

Forward, 5’-ggggacaagtttgtacaaaaaagcaggcttagtatgttgggttattaagtgatttgacc-3’

Reverse, 5’-ggggacaacttttgtatacaaagttgtcgaagagatgtcaaaattaaaatagcctg-3’

We subsequently cloned p*Rbp4* into pDONR 221 P1-P5r (Invitrogen) using BP clonase (ThermoFisher Scientific, 11789020) to generate pENTR-L1-*pRbp4*-R5. Successful insertion of p*Rbp4* was verified by test digestion and sequencing. We then performed an LR reaction (LR clonase; ThermoFisher Scientific, 12538120) with pENTR-L1-*pRbp4*-R5, pENTR-L5-*Gal4*-L2 (Addgene #32304, deposited by Steve Stowers), and pDESTsvaw (Addgene #32318, deposited by Steve Stowers) to generate pDESTsvaw-*pRbp4-Gal4*, where *pRbp4* is upstream of Gal4. pDESTsvaw contains a *mini-white* rescue construct, which enables transgenic animal selection. We verified that *pRbp4* and *Gal4* were properly inserted via test digestion and sequencing. Injection and selection of transgenic flies was performed by GenetiVision (Houston, TX). Embryos lacking the *mini-white* gene (w-) were injected with the pDESTsvaw-*pRbp4-Gal4* plasmid and, using PhiC31 integration, pDESTsvaw-*pRbp4-Gal4* was inserted on the third chromosome at docking site VK27. Transgenic flies were selected by the presence of red eyes (*mini-white* gene rescue) and balanced over TM6B, a third chromosome balancer, using standard crossing procedures to generate a stable line. The pENTR-L1-*pRbp4*-R5 and pDEST-*pRbp4-Gal4* plasmids are available upon request.

### Immunostaining, microscopy and image processing

Testes were dissected in 1X phosphate-buffered saline (PBS; 137 mM NaCl, 2.7 mM KCl, 10 mM Na_2_HPO_4_, 1.8 mM KH_2_PO_4_) and then immediately fixed in 4% paraformaldehyde diluted in PBS. Testes were washed three times in PBT (1X PBS, 0.1% Tween-20), then incubated in blocking buffer (3% BSA in 1X PBS) for at least 1 h at room temperature. Testes were incubated with the primary antibody diluted in blocking buffer containing 2% Triton X-100 overnight at 4°C. The next day, testes were washed five times with PBT prior to applying the secondary antibody. Testes were incubated with the secondary antibody for at least 3 h at room temperature in the dark. After the secondary antibody solution was removed, testes were washed five times with PBT. 1 µM Hoechst 33342 was incubated in the first wash to stain DNA. Testes were mounted in Vectashield antifade mounting medium prior to imaging.

The following antibodies were used in this study: rabbit anti-GFP (1:1000; Invitrogen A21311), rabbit anti-H2Aub (1:100; CST #8240S) and goat anti-rabbit 488 (1:500; Invitrogen A11034).

Images were acquired using an inverted Leica SP8 confocal microscope, equipped with a 40× objective (NA 1.30) and a white-light laser. Images were processed using Leica LAS X software, and quantifications were performed using Fiji (NIH) on 8-bit images.

### VCP-GFP quantification

We used Fiji (NIH) to quantify VCP-GFP intensity in the nucleus and cytosol of germ cells at various stages of spermatogenesis (see below for stage identification information). Briefly, we outlined the nucleus and a small region within the cytosol in five cells per stage per testis and measured the mean fluorescence intensity. This was done for 10 testes total for each condition. VCP-GFP was imaged using a 488 nm excitation laser and a Hybrid detector set at 500–550 nm. The excitation laser was set at 2.5% for all testes imaged with gain left at its default level, such that GFP intensity could be reliably compared between genotypes. For presentation purposes, the mean intensity of the control group was normalized to one.

### Fertility assay

Single male flies were placed in a vial with 2–3 wild type (w^1118^) virgin females shortly after eclosion. Males were transferred to a fresh vial with new wild type virgin females 2–3 d later. The presence or absence of progeny was scored after each mating. Males that failed to produce progeny in both matings were scored as infertile. Males that were able to produce progeny in at least one mating were scored as fertile. Flies were kept at 25°C on standard cornmeal/agar food for all matings.

### Phalloidin staining

Phalloidin staining was performed by fixing testes in 4% paraformaldehyde immediately following dissection. Testes were then washed three times in PBT (1X PBS, 0.1% Tween-20). After washing, testes were incubated in 1:400 phalloidin (Invitrogen A12380) for 1 h at room temperature in the dark. Following staining, testes were washed three times in PBT (1X PBS, 0.1% Tween-20). 1 µM Hoechst 33342 was incubated in the first wash to stain DNA. Testes were mounted in Vectashield antifade mounting medium prior to imaging.

### Germ-cell stage identification

Germ-cell staging was performed primarily based on chromatin morphology. Spermatocytes were identified based on chromatin features described in [21]. Spermatids were identified based on chromatin and mitochondrial features described in [4].

### Statistical analyses

Information on sample size and statistics is provided in figure legends where applicable. Data normality was tested via the D’Agostino-Pearson normality test in combination with Q–Q plots prior to performing follow-up statistical analyses using GraphPad Prism software. Statistical tests used to determine significance are indicated in figure legends. A student’s unpaired t-test was used when unpaired data for two groups were normally distributed and standard deviation was equal between groups. Welch’s unpaired t-test was used when unpaired data for two groups were normally distributed but standard deviation was not equal. The Mann–Whitney U-test was used when unpaired data for two groups were not normally distributed.

## Results

### VCP is expressed ubiquitously throughout the male *Drosophila* germline

VCP serves critical functions in the development of both spermatogonia [2] and spermatocytes [3]. However, whether VCP is also expressed and performs important, germline-specific functions in later stages of spermatogenesis, such as the spermatid stage, has not been explicitly explored. Using a VCP-GFP fly line, where a GFP tag was inserted at the C-terminus of the endogenous *VCP* locus via CRISPR-Cas9 [15], we noticed that VCP is expressed throughout the male germline (Figure 1b). As previously described [3], VCP is cytosolic in spermatogonia (Figure 1c) but enters the nucleus as cells transition into the spermatocyte stage (Figure 1c). Notably, we also observed bright VCP-GFP in the nucleus of round spermatids and canoe-stage spermatids (Figure 1c). This suggests that VCP is subject to developmental regulation at multiple stages, and it may serve some function in spermatid nuclei.

### Rbp4-Gal4 drives transgene expression in germ cells after mitotic stages of spermatogenesis but not earlier

**Figure 2.**
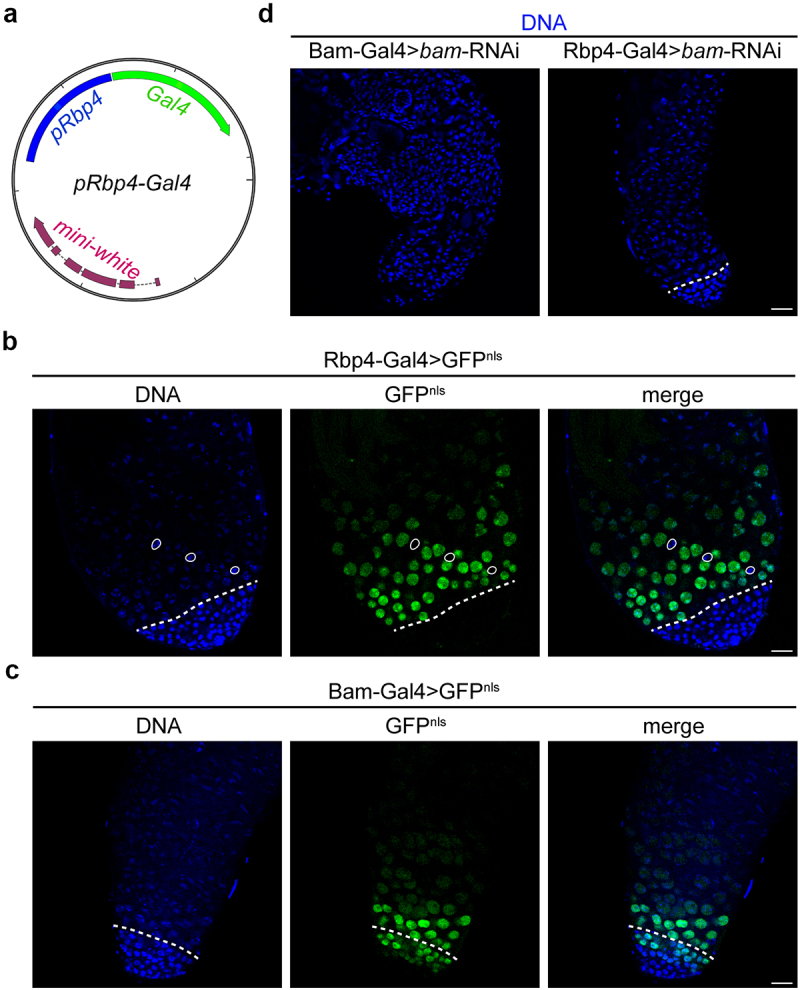
Rbp4-Gal4 drives transgene expression in spermatocytes but not spermatogonia. (a) Map of the Rbp4-Gal4 plasmid. Blue indicates the *Rbp4* promoter, green indicates the *Gal4* coding sequence, and red indicates the *mini-white* gene (selection marker). (b) Images of Hoechst (DNA) and Rbp4-Gal4-driven GFP^nls^ in a *Drosophila* testis. The dashed line indicates the spermatogonia-to-spermatocyte transition. Example cyst-cell nuclei are outlined. (c) Images of Hoechst (DNA) and Bam-Gal4-driven GFP^nls^ in a *Drosophila* testis. The dashed line indicates the spermatogonia-to-spermatocyte transition. (d) Images of Hoechst (DNA) in Bam-Gal4>*bam*-RNAi and Rbp4-Gal4>*bam*-RNAi testes. Note that chromatin is compact and cells are small throughout Bam-Gal4>*bam*-RNAi testes, indicative of spermatogonia, while the majority of cells present in Rbp4-Gal4>*bam*-RNAi testes are larger and exhibit tri-lobed chromatin (paired bivalents), characteristic of spermatocytes. The dashed line indicates the spermatogonia-to-spermatocyte transition. Bars, 20 µm. See also Figure S1.

Given this localization pattern, we aimed to develop a Gal4/UAS strain that would allow us to probe possible VCP functions in the spermatid stage. As noted previously, the three major germline-specific Gal4 drivers (Vasa-Gal4, Nanos-Gal4 and Bam-Gal4) initiate transgene expression at the GSC and/or spermatogonia stages [8]. Because a germline-specific Gal4 driver capable of driving transgene expression starting at the spermatocyte stage, but not at earlier stages, currently does not exist, we sought to generate a germline-specific Gal4 driver that activates in the early spermatocyte stage, as this could permit the study of gene function during later stages of spermatogenesis. We reasoned that a Gal4 driver of this nature must meet the following criteria: 1) the driver must be under control of a promoter that is inactive or minimally active in spermatogonia; 2) the promoter must be highly active in spermatocytes, particularly at early stages; and 3) the promoter should be minimally active in somatic cells to prevent potential embryonic lethality and unintended physiological effects. To identify the gene promoters that fit these criteria, we examined *Drosophila* transcriptomic datasets that distinguish genes based on germline versus somatic expression [18,19] and expression at different germ-cell stages [13,14]. This analysis yielded several genes, but we prioritized genes that had been experimentally shown to be expressed and to function in the male germline (Fig. S1; see Methods). We found that *Rbp4* fit our criteria the best; not only is *Rbp4* lowly expressed in spermatogonia (Fig. S1) [13,14] and minimally expressed outside of the germline [18,19] but, among the candidates, it showed the highest early-spermatocyte expression (Fig. S1) [14]. We thus cloned the promoter of *Rbp4* upstream of the *Gal4* coding sequence (Figure 2a) and generated Rbp4-Gal4 transgenic flies.

We tested the specificity of transgene expression under Rbp4-Gal4 control by driving nuclear-localized GFP (GFP^nls^) expression. For comparison, we also examined GFP^nls^ expression under the control of Bam-Gal4. Rbp4-Gal4-driven GFP^nls^ signal was absent from mitotic spermatogonia but clearly visible in spermatocytes (Figure 2b). In contrast, Bam-Gal4-driven GFP^nls^ signal was present in late spermatogonia stages, as well as in spermatocytes (Figure 2c). Thus, Rbp4-Gal4 activates developmentally later than Bam-Gal4. Importantly, Rbp4-Gal4-driven GFP^nls^ signal was undetectable in somatic cyst-cell nuclei (Figure 2b, outlines), indicating that Rbp4-Gal4 activity is restricted in the testis to the germline. To further verify that Rbp4-Gal4 is not active in spermatogonia, we knocked down *bag of marbles* (*bam*), which is required for the spermatogonia-to-spermatocyte transition [22]. Whereas Bam-Gal4-driven knockdown of *bam* blocked the spermatogonia-to-spermatocyte transition, as indicated by an absence of spermatocytes (Figure 2d, left panel), Rbp4-Gal4-driven knockdown of *bam* did not affect developmental progression to the spermatocyte stage (Figure 2d, right panel). Collectively, these data indicate that Rbp4-Gal4 activity is restricted in the testis to germ cells and becomes active at the spermatocyte stage.

### Rbp4-Gal4 drives efficient knockdown of endogenous *VCP* in spermatocytes and spermatids

We next assessed the usefulness of this tool in manipulating gene expression in post-spermatogonial stages of male germline development. We recently found that *VCP* knockdown using the Bam-Gal4 driver arrested cells at the spermatocyte stage, when VCP normally redistributes from the cytosol to the nucleus (Figure 1c) [3]. Because Bam-Gal4 activates in spermatogonia, whereas Rbp4-Gal4 activates in the spermatocyte stage, we reasoned that *VCP* knockdown using this new driver may produce a different phenotype; the later-stage knockdown may not activate in time to produce the initial degree of knockdown required to block spermatocyte differentiation, but it might reveal later defects.

**Figure 3.**
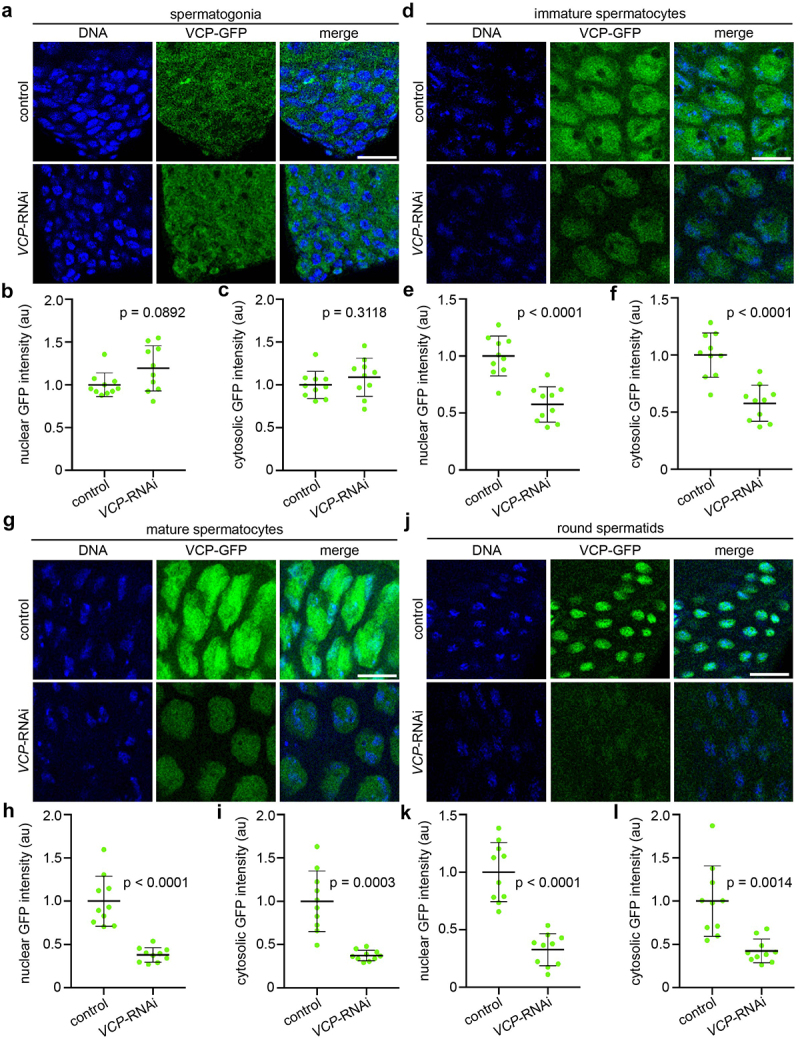
Rbp4-Gal4 drives significant knockdown of *VCP* expression in germ cells after mitotic stages of spermatogenesis. (a, d, g, j) Images of Hoechst (DNA) and VCP-GFP in spermatogonia (a), immature spermatocytes (d), mature spermatocytes (g), and round spermatids (j) of control (Rbp4-Gal4/+) and *VCP*-RNAi (Rbp4-Gal4>*VCP*-RNAi) testes. Bars, 20 µm. (b, e, h, k) Quantification of VCP-GFP intensity in nuclei of spermatogonia (Mann-Whitney U-test, U = 27; b), immature spermatocytes (unpaired t-test, *t* = 5.762, df = 18; e), mature spermatocytes (Welch’s t-test, *t* = 6.507, df = 10.49; h), and round spermatids (Welch’s t-test, *t* = 7.311, df = 13.9; k) of control (Rbp4-Gal4/+) and *VCP*-RNAi (Rbp4-Gal4>*VCP*-RNAi) testes. (c, f, i, l) Quantification of VCP-GFP intensity in the cytosol of spermatogonia (unpaired t-test, *t* = 1.041, df = 18; b), immature spermatocytes (unpaired t-test, *t* = 5.328, df = 18; e), mature spermatocytes (Welch’s t-test, *t* = 5.594, df = 9.543; h), and round spermatids (Welch’s t-test, *t* = 4.240, df = 11.04; k) of control (Rbp4-Gal4/+) and *VCP*-RNAi (Rbp4-Gal4>*VCP*-RNAi) testes. Mean ± s.D. *n* = 10 testes of each genotype for each graph. *p-*values are indicated on all graphs.

As a first step to evaluate *VCP*-RNAi using Rbp4-Gal4, we quantified endogenous VCP-GFP fluorescence intensities at different stages of spermatogenesis. As expected, VCP-GFP fluorescence intensity was not affected in spermatogonia of *VCP-*RNAi testes compared to controls (Figure 3a-c). In contrast, VCP-GFP signal was substantially reduced by Rbp4-Gal4-driven *VCP*-RNAi at later stages of male germ-cell development (Figure 3d-l). Moreover, unlike testes with *VCP*-RNAi driven by Bam-Gal4, testes with *VCP*-RNAi driven by Rbp4-Gal4 produced post-meiotic round spermatids (Figure 3j), consistent with a later timepoint of gene knockdown and inhibition. These data indicate that Rbp4-Gal4 is indeed capable of knocking down *VCP* expression in spermatocytes and round spermatids, and that Rbp4-Gal4-driven knockdown of *VCP* permits development to the spermatid stage, providing an opportunity to experimentally assess potential *VCP* functions in later stages of spermatogenesis.

### VCP regulates spermatid chromatin condensation and individualization

**Figure 4.**
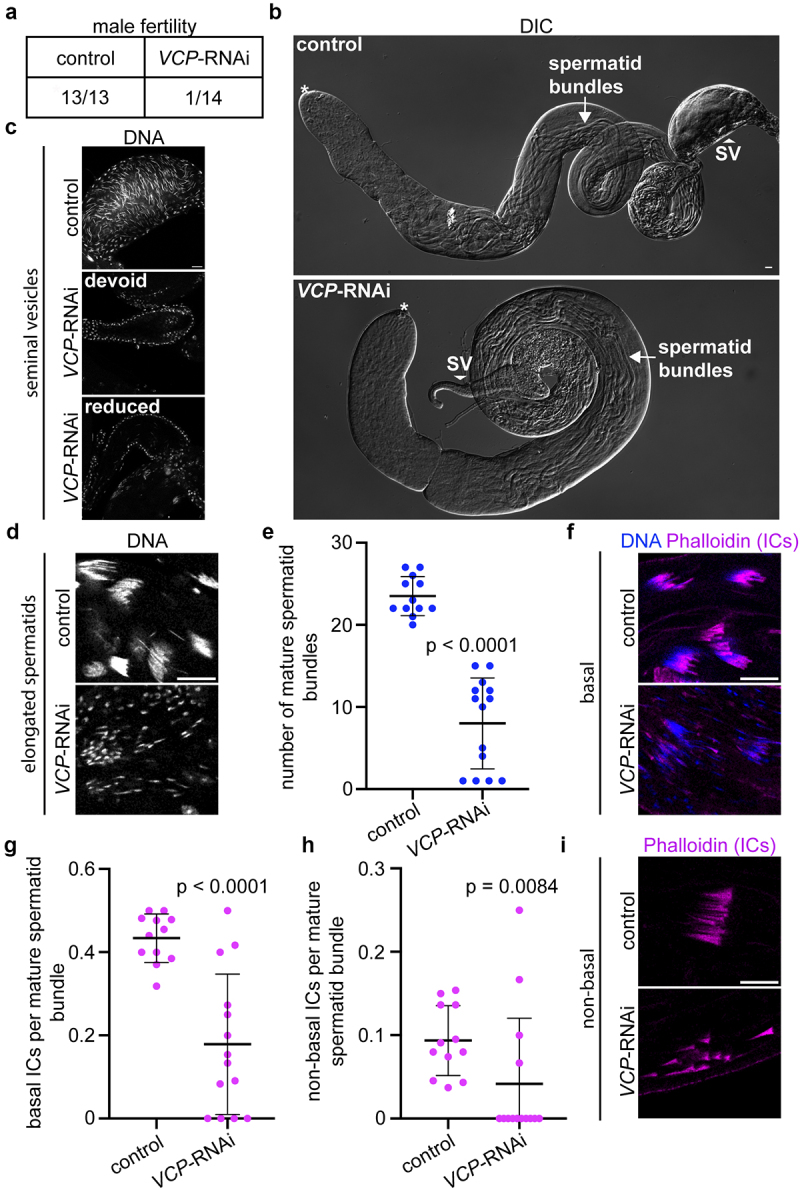
VCP promotes elongated spermatid chromatin condensation and spermatid individualization. (a) Table of the proportion of males that were fertile for each genotype. (b) DIC images of whole control (Rbp4-Gal4/+) and *VCP*-RNAi (Rbp4-Gal4>*VCP*-RNAi) testes. The arrows indicate elongated spermatid bundles. The arrowheads indicate the seminal vesicles (SV), where mature sperm are stored. The asterisks indicate the apical tips of the testes, where spermatogenesis initiates. (c) Images of Hoechst (DNA)-labelled seminal vesicles of control (Rbp4-Gal4/+) and *VCP*-RNAi (Rbp4-Gal4>*VCP*-RNAi) testes. Needle-shaped DNA indicates mature sperm. (d) Images of Hoechst (DNA)-labelled elongated spermatid nuclei located at the basal end of control (Rbp4-Gal4/+) and *VCP*-RNAi (Rbp4-Gal4>*VCP*-RNAi) testes. (e) Quantification of the number of mature elongated spermatid bundles in control (Rbp4-Gal4/+; *n* = 12) and *VCP*-RNAi (Rbp4-Gal4>*VCP*-RNAi; *n* = 14) testes. Mean ± s.D. Mann-Whitney U-test (U = 0). (f) Images of Hoechst (DNA) and Phalloidin (ICs) at the basal ends of control (Rbp4-Gal4/+) and *VCP*-RNAi (Rbp4-Gal4>*VCP*-RNAi) testes. (g) Quantification of the number of ICs per mature elongated spermatid bundle at the basal end of the testis in control (Rbp4-Gal4/+; *n* = 12) and *VCP*-RNAi (Rbp4-Gal4>*VCP*-RNAi; *n* = 14) testes. Mean ± s.D. Welch’s t-test (*t* = 5.296, df = 16.49). (h) Quantification of the number of ICs per mature elongated spermatid bundle at the non-basal end of the testis in control (Rbp4-Gal4/+; *n* = 12) and *VCP*-RNAi (Rbp4-Gal4>*VCP*-RNAi; *n* = 14) testes. Mean ± s.D. Mann-Whitney U-test (U = 35). (**I**) Images of Phalloidin-labelled non-basal ICs in control (Rbp4-Gal4/+) and *VCP*-RNAi (Rbp4-Gal4>*VCP*-RNAi) testes. Bars, 20 µm. *p-*values are indicated on all graphs.

Though the use of Rbp4-Gal4 to drive *VCP-*RNAi evaded the meiotic-arrest phenotype observed when using Bam-Gal4, male flies were still largely infertile (Figure 4a), suggesting a critical function for VCP in post-meiotic stages of spermatogenesis. Interestingly, Rbp4-Gal4-driven *VCP*-RNAi testes appeared morphologically similar to controls, and elongated spermatids were observable by DIC (Figure 4b). However, seminal vesicles, which store mature sperm, appeared smaller in Rbp4-Gal4-driven *VCP*-RNAi testes compared controls (Figure 4b) and most seminal vesicles of Rbp4-Gal4 *VCP*-RNAi testes were completely devoid of sperm (Figure 4c, middle panel) though some retained a few sperm (Figure 4c, bottom panel).

Given the apparent defect in mature sperm formation in Rbp4-Gal4 *VCP*-RNAi testes, we hypothesized that *VCP* may support maturation of elongated spermatids and/or spermatid individualization, the terminal differentiation event of spermatogenesis [4]. To test the first possibility, we analysed chromatin condensation, which is tightly linked to spermatid maturation [4]. If *VCP* were to promote spermatid maturation, one would expect to observe incompletely condensed spermatid chromatin in *VCP*-RNAi testes. Indeed, we found that elongated spermatid chromatin failed to completely condense upon *VCP* knockdown (Figure 4d). This phenotypic abnormality was accompanied by a significant reduction in the number of mature elongated spermatids (Figure 4e). Thus, we concluded that one germline-specific function of *VCP* during late spermatogenesis is to promote elongated spermatid chromatin condensation.

We next tested whether *VCP* might additionally regulate spermatid individualization. During the individualization process, filamentous actin (F-actin)-based investment cones form near the nucleus of each of the 64 elongated spermatids within a cyst at the basal end of the testis. Collectively, these investment cones make up a complex known as the individualization complex (IC) [4]. Once ICs have fully formed, ICs synchronously translocate down spermatid tails in a basal to apical direction to remove excess cytoplasm and organelles [4]. To test if *VCP* promotes proper spermatid individualization, we labelled ICs with an F-actin dye, phalloidin, in control and *VCP*-RNAi testes. Because we had observed a reduction in the number of mature spermatid bundles (Figure 4e), which would not form ICs, and would thus generate confounding results, we normalized the number of ICs to the total number of mature spermatid bundles present. Consistent with a defect in spermatid individualization, we observed a significant reduction in the number of ICs per mature spermatid bundle at the basal end of the testis in *VCP*-RNAi testes compared to controls (Figure 4f,). We likewise observed a similar reduction in the number of non-basal ICs (i.e. those that had begun movement to the apical end) per mature spermatid bundle in *VCP*-RNAi testes compared to controls (Figure 4h). Notably, of the few non-basal ICs we did see in *VCP*-RNAi testes, we found that they often moved asynchronously (Figure 4i). Overall, these data indicate that *VCP* plays an additional role in the formation and synchronous movement of ICs to support proper spermatid individualization.

### VCP promotes the histone-to-protamine transition

**Figure 5.**
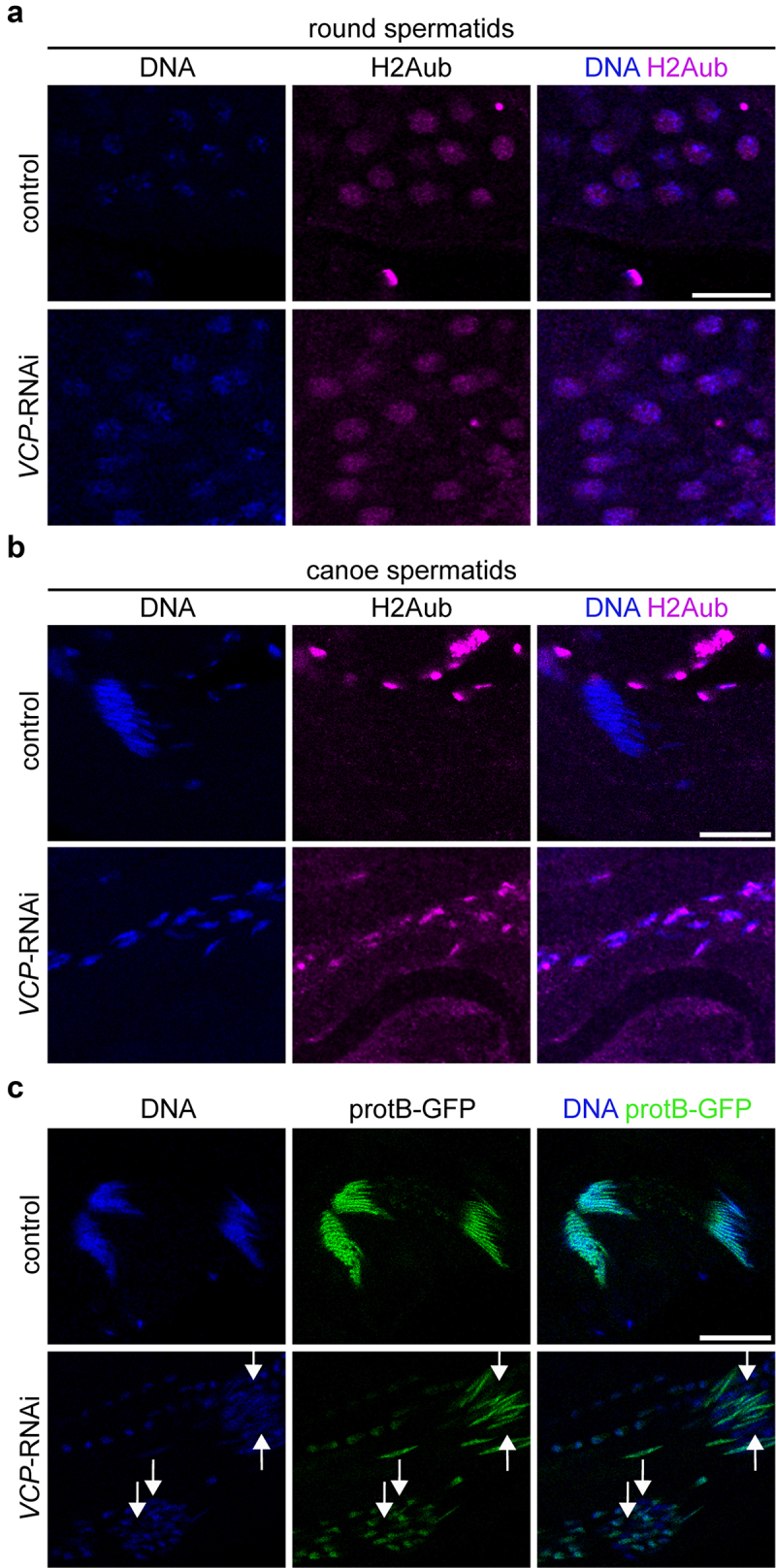
VCP promotes H2Aub clearance and protamine incorporation in spermatids. (a) Images of Hoechst (DNA) and H2Aub in round spermatids of control (Rbp4-Gal4/+) and *VCP*-RNAi (Rbp4-Gal4>*VCP*-RNAi) testes. (b) Images of Hoechst (DNA) and H2Aub in canoe-stage spermatids of control (Rbp4-Gal4/+) and *VCP*-RNAi (Rbp4-Gal4>*VCP*-RNAi) testes. (c) Images of Hoechst (DNA) and protamine B (protB-GFP) in canoe-stage spermatids of control (Rbp4-Gal4/+) and *VCP*-RNAi (Rbp4-Gal4>*VCP*-RNAi) testes. The arrows indicate example nuclei that failed to incorporate protB-GFP. Bars, 20 µm.

As spermatids mature, histones are replaced by protamines to drive hyper-condensation of chromatin [5,6]. Interestingly, mono-ubiquitinated H2A (H2Aub), which is downregulated in spermatocytes by VCP [3], is cleared from round spermatid nuclei just prior to the histone-to-protamine transition [6]. Because nuclei fail to properly condense in *VCP*-RNAi testes, we were curious whether H2Aub dynamics in spermatids may also be affected by knockdown of *VCP*. In control testes, we observed that H2Aub was present in round spermatid nuclei (Figure 5a) and absent from canoe-stage spermatids (Figure 5b), as previously demonstrated [6]. In *VCP*-RNAi testes, H2Aub was present in round spermatids at comparable levels to controls (Figure 5a). However, H2Aub was retained in canoe-stage spermatids of *VCP*-RNAi testes (Figure 5b).

Because clearance of H2Aub from spermatid nuclei has been postulated to be a critical step for the histone-to-protamine transition in flies [6], we hypothesized that protamine expression and/or incorporation may be affected by the absence of VCP. We tested this hypothesis by imaging a GFP-tagged protamine (protB-GFP) that was expressed from its endogenous promoter. While we did observe protB-GFP expression in some *VCP*-RNAi canoe-stage spermatids (Figure 5c), we noted that protB-GFP was not uniformly expressed in all spermatid nuclei; strikingly, protB-GFP signal was present in some nuclei, but absent from adjacent nuclei within the same cyst (Figure 5c, arrows). Collectively, our data suggest that VCP affects the robustness of the histone-to-protamine transition, possibly by controlling H2Aub levels, and this function likely in turn supports chromatin condensation and spermatid maturation.

## Discussion

Previous studies have used the *β2-tubulin* promoter to express transgenes in the late stages of spermatogenesis [23–31]. However, attempts to generate a late-stage Gal4 driver under the control of the *β2-tubulin* promoter have not been fruitful [9], likely because the promoter becomes active too late in the spermatocyte stage to produce a sufficient amount of Gal4 to drive transgene expression. In this study, we have overcome this obstacle by generating Rbp4-Gal4, which activates early in the spermatocyte stage. As a proof-of-principle, we used Rbp4-Gal4 to successfully knock down *VCP* in late spermatogenesis, providing information on late-stage functions of VCP in spermatogenesis.

In theory, the Rbp4-Gal4 driver could also be applied to study the function of virtually any other gene that regulates the development of spermatids, or maybe even late-stage spermatocytes. Recent single-cell RNA sequencing studies in *Drosophila* [13,19,32,33] and a stage-specific *Drosophila* spermatogenesis proteomics study [34] have provided robust datasets that may suggest important functions for particular genes in the late stages of spermatogenesis. However, some of these genes may also serve important functions in the early stages of spermatogenesis. In the past, this would have posed an issue in studying the function of these genes in later stages, as had been the case for *VCP* [2,3]. As demonstrated in our study, Rbp4-Gal4 permits the investigation of potential pleiotropic gene functions in the spermatogenesis program, which provides an opportunity to advance this field of study. Notably, because UAS-RNAi lines are readily available from various stock centres (BDSC, VDRC, Kyoto, etc.), the Rbp4-Gal4 driver can now be combined with gene knockdown experiments and even screens to rapidly identify genes that function in the late stages of spermatogenesis.

Consistent with this driver activating at the spermatocyte stage, Rbp4-Gal4 sufficiently knocked down *VCP* in spermatocytes and spermatids (Figure 3). Although *VCP*-RNAi using Rbp4-Gal4 decreased VCP levels in immature and mature spermatocytes, it did not lead to a developmental arrest at the spermatocyte stage, as had *VCP*-RNAi when driven by Bam-Gal4 [3]. One important point to note is that Rbp4-Gal4 becomes active developmentally later than Bam-Gal4, which activates in spermatogonia (Figure 2b, c). It could be that *VCP* knockdown must be initiated at the spermatogonia-to-spermatocyte transition to cause an arrest at the spermatocyte stage, and that Rbp4-Gal4, while active in spermatocytes, activates too late to produce this arrest. These small differences in timing in fact enabled us to probe *VCP* function later in spermatogenesis using Rbp4-Gal4, which was previously not possible using available Gal4 drivers.

Currently, there are some open questions regarding how *VCP* molecularly supports spermatid development. We previously found that the knockdown of *VCP* using Bam-Gal4 decreases the expression of *mst35Bb* [3], which encodes protB [5]. While we detected protB in some spermatid nuclei upon *VCP* knockdown using Rbp4-Gal4 (Figure 5c), protB was evidently not incorporated in all spermatid nuclei following this inhibition (Figure 5c), consistent with a defect in the histone-to-protamine transition. Additionally, H2Aub, which restricts protein access to DNA and histones [35], was not properly downregulated in canoe-stage spermatids of *VCP*-RNAi testes (Figure 5b). The failure to downregulate H2Aub could impede the insertion of protamines by restricting access of transition proteins, as has been previously proposed [6,36]. Further, a recent study identified *mst77Y* as a dominant-negative regulator of protamine incorporation [29]; overexpression of *mst77Y* causes spermatid chromatin condensation and protamine incorporation defects, which are similar to the phenotypes observed in *VCP*-RNAi testes. In the future, it will be important to determine whether blocking H2Aub in spermatids that lack *VCP* function is sufficient to support chromatin condensation and to test whether VCP regulates *mst77Y* expression.

In this study, we also found Rbp4-Gal4-driven knockdown of VCP disrupts IC formation and synchronous IC translocation down spermatid tails (Figure 4d-1). Notably, these phenotypes are reminiscent of those observed in caspase mutants [26,37]. It is possible that after spermatid maturation, VCP may act in the caspase activation pathway to control IC formation and translocation. In support of this hypothesis, our previous study demonstrated that VCP supports the expression of *cyt-c-d* [3], which is required for caspase activation and proper spermatid individualization [37]. Another possibility is that VCP could indirectly activate caspases, similar to its function in the dendritic pruning process [38]. Going forward, it will be interesting to test how VCP functions in the germline to promote proper spermatid individualization, which will now be facilitated by the newly generated Rbp4-Gal4 tool.

## Supplementary Material

Supplemental Material

## Data Availability

All the data associated with this manuscript are provided in the paper. Additional information and original image data are available from the corresponding authors upon request.
