# Supplementary figures and images for: Rbp4-Gal4, a germline driver that activates in meiosis, reveals functions for VCP in spermatid development
Source: Fly (Austin). 2023 Jul 12;17(1):2234795. doi: 10.1080/19336934.2023.2234795 (PMC10339771; doi:10.1080/19336934.2023.2234795)

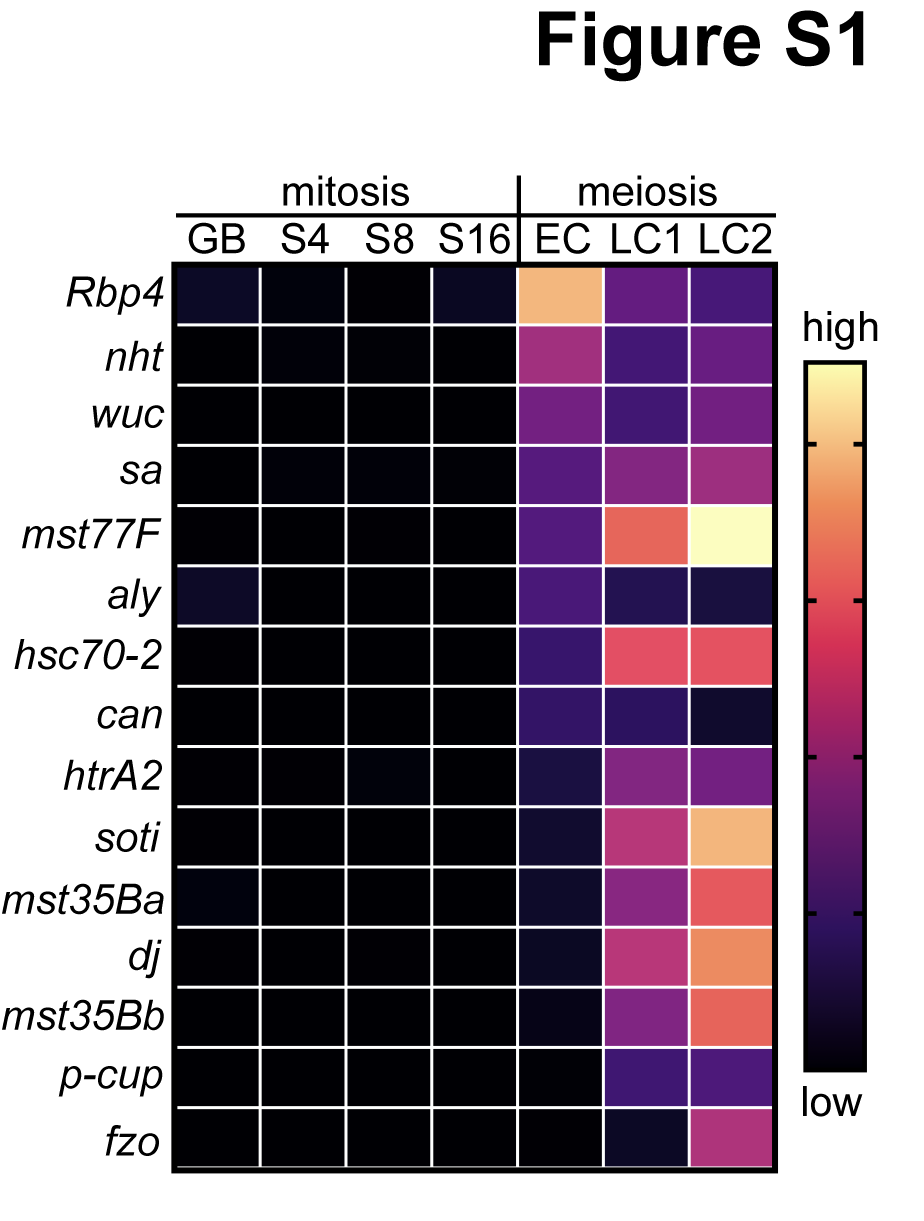

Supplement: Supplemental Material [file KFLY_A_2234795_SM0162.tif]
